# No Polymorphism in* Plasmodium falciparum K13* Propeller Gene in Clinical Isolates from Kolkata, India

**DOI:** 10.1155/2015/374354

**Published:** 2015-11-25

**Authors:** Moytrey Chatterjee, Swagata Ganguly, Pabitra Saha, Biswabandhu Bankura, Nandita Basu, Madhusudan Das, Subhasish K. Guha, Ardhendu K. Maji

**Affiliations:** ^1^Protozoology Unit, Department of Microbiology, Calcutta School of Tropical Medicine, 108 Chittaranjan Avenue, Kolkata 700 073, India; ^2^Department of Microbiology, NRS Medical College, 138 AJC Bose Road, Kolkata 700 014, India; ^3^Department of Zoology, APC Roy Government College, Himachal Bihar, Matigara, Siliguri, West Bengal 734 010, India; ^4^Department of Zoology, Ballygunge Science College, University of Calcutta, 35 Ballygunge Circular Road, Kolkata 700 019, India; ^5^Calcutta School of Tropical Medicine, 108 Chittaranjan Avenue, Kolkata 700 073, India; ^6^Department of Tropical Medicine, Calcutta School of Tropical Medicine, 108 Chittaranjan Avenue, Kolkata 700 073, India

## Abstract

Molecular markers associated with artemisinin resistance in* Plasmodium falciparum* are yet to be well defined. Recent studies showed that polymorphisms in* K13* gene are associated with artemisinin resistance. The present study was designed to know the pattern of polymorphisms in propeller region of* K13* gene among the clinical isolates collected from urban Kolkata after five years of ACT implementation. We collected 59 clinical isolates from urban Kolkata and sequenced propeller region of* K13* gene in 51 isolates successfully. We did not find any mutation in any isolate. All patients responded to the ACT, a combination of artesunate + sulphadoxine-pyrimethamine. The drug regimen is still effective in the study area and there is no sign of emergence of resistance against artemisinin as evidenced by wild genotype of* K13 *gene in all isolates studied.

## 1. Introduction

Emergence of resistance against different antimalarial drugs (AMDs) particularly in* Plasmodium falciparum *is a serious problem in combating malaria. During the 1990s* P. falciparum* acquired resistance to chloroquine (CQ) and sulphadoxine-pyrimethamine (SP) throughout all malaria endemic countries. World Health Organization (WHO) recommended artemisinin combination therapy (ACT) as first line agent to treat all uncomplicated* falciparum* malaria throughout the world. After introduction of the ACTs and increased use of insecticide treated bed nets (ITBNs), the mortality and morbidity due to* falciparum* malaria reduced globally [1] including India [2, 3]. Unfortunately, within a few years* P. falciparum* developed resistance against artemisinin. Artemisinin resistance is defined by longer parasite clearance time in in vivo efficacy study and in in vitro ring stage survival assay [4–6]. Artemisinin resistance was first reported from Western Cambodia [7, 8] in 2009. Now it has spread throughout Southeast Asian countries [6, 9–13].

ACT, a combination of artesunate + sulphadoxine-pyrimethamine (AS + SP), was first introduced in India in 2009 to treat all uncomplicated* falciparum* cases. Previously ACT was recommended only in some high risk districts like Purulia and Jalpaiguri of West Bengal. Several efficacy studies showed that this combination is still effective in various parts of the country [14, 15] except in northeastern part [13] where AS + SP has been replaced by artemether + lumefantrine. Antimalarial drug efficacy monitoring every two years can predict the emergence of drug resistance, if any. Though in vivo therapeutic efficacy study remains the gold standard for the purpose, molecular markers have also been validated as alternative tool. A single mutation, K76**T** in* pfcrt* gene, is a hallmark of CQ resistance [16, 17] and a quintuple mutation in* pfdhfr *and* pfdhps *genes strongly correlated with SP resistance [18]. Recently polymorphisms in the propeller region of* P. falciparum* Kelch protein (*K13* gene) have been found to be associated with artemisinin resistance [19]. Polymorphisms in* K13* gene have been reported from different countries [9, 19–26] but such report from India is scarce. Recently a report showed that mutations in* K13* gene are limited [27] in the strains isolated from northeastern part of the country. The present study deals with the pattern of polymorphisms in propeller region of* K13* gene among the clinical isolates collected from Kolkata, India, after five years of ACT implementation.

## 2. Materials and Methods

### 2.1. Study Site and Collection of Blood Samples

The study was conducted at the Malaria Clinic attached to Protozoology Unit of the Calcutta School of Tropical Medicine, Kolkata, from January to December, 2014. In routine practice diagnosis was done by microscopy following Giemsa staining of thick and thin blood films. Report and medicines were distributed on the same day. All treated* P. falciparum* cases were advised to attend the clinic for reexamination on days 1, 2, 3, 7, 14, 21, 28, 35, and 42. Two to three mL EDTA blood samples were collected from the microscopically* P. falciparum *positive patients during distribution of report and medicine after obtaining informed consent or assent from the patients/guardians of the children below 14 years of age. The study protocol was reviewed and cleared by Institutional Ethics Committee of the Calcutta School of Tropical Medicine, Kolkata.

### 2.2. DNA Isolation

*P. falciparum *genomic DNA was isolated from whole blood samples using QIAamp DNA blood mini kit (Qiagen, Hilden, Germany), following the manufacturer's instructions. Extracted DNA of all the samples was preserved at −20°C and an aliquot was used as the DNA source for further study.

### 2.3. Nested PCR and Sequencing of* K13* Gene Propeller Region

The propeller domain of* K13* gene was amplified by nested PCR method as described by Ariey et al., 2014. All amplification reactions were carried out in a final volume of 20 *μ*L for primary PCR and 50 *μ*L for nested PCR. The oligonucleotide primers and PCR conditions are summarized in Table 1.

The quality and concentration of PCR products were ascertained by 1.5% agarose gel electrophoresis following ethidium bromide stain. The nested PCR products were purified using QIAquick PCR Purification Kit (Qiagen). Sequencing was outsourced from Amnion Biosciences Pvt. Ltd., Bangalore, India.

### 2.4. Analysis of Sequence

The sequences were analyzed using the free software Bioedit Sequence Alignment Editor version 7.0.5.2 and aligned with sequences of PF3D7_1343700 Kelch protein propeller domain using the online sequence alignment tool ClustalW.

## 3. Results

During the study period a total of 8877 febrile patients attended the clinic for diagnosis and treatment for malaria. Among them 1223 were positive for* P. vivax* and 107 were positive for* P. falciparum*. The presence of mixed infection with both* P. vivax* and* P. falciparum *cannot be ruled out as a confirmatory molecular method was not performed. Out of 107* P. falciparum* cases blood samples were collected from 59 patients who consented. Though it was not a therapeutic efficacy study all treated patients were followed up both clinically and parasitologically as mentioned earlier. It was found that all 59 patients responded to AS + SP therapy, that is, no case of recrudescence recorded during six-week posttreatment period.

Out of 59 samples, sequencing of propeller region of* K13* gene was done in 51 isolates. After alignment with PF3D7_1343700, it was found that all 51 sequences were perfectly aligned from nucleotide number 1327 to 1998 (codons 443–666), that is, from blade 1 to blade 5 of the propeller region of* K13* gene. In blade 6 sequences were studied partly. All the strains were wild type having no polymorphism in this region of the gene. The nucleotide sequence of* K13* gene of* P. falciparum* isolate from the study site has been submitted in GenBank under Accession number KR779827.1.

## 4. Discussion

Unlike chloroquine and sulphadoxine-pyrimethamine resistance, artemisinin resistance in* P. falciparum* is yet to be well defined as definite phenotypes have not been well established except delayed parasite clearance following chemotherapy and ring stage survival assay (RSA) [7, 9]. These parameters are indications of emergence of resistance by the parasites against artemisinin derivatives. WHO [28] defined suspected partial artemisinin resistance as follows: persistent parasitemia by microscopy on day 3 after treatment with ACT/artesunate monotherapy in 10%/more patients or presence of resistance-associated mutations in* K13* gene in at least 5% of patients or parasite clearance half-life is more than 5 hours after treatment. Confirmed partial artemisinin resistance is defined as presence of* K13* resistance-associated mutations in at least 5% of patients to have either persistent parasitaemia by microscopy on day 3 or a parasite clearance half-life of ≥5 hours after treatment with ACT or artesunate monotherapy. To conserve the efficacy of artemisinin derivatives, monotherapy with this drug has been stopped and is recommended with another long acting partner drug. So efficacy study of any ACT does not deal with efficacy of artemisinin alone. As definite phenotype of ACT resistance is very rare, the association of polymorphisms in marker genes is very difficult to correlate with efficacy outcome. Ariey et al., 2014 [19], have recently shown that four polymorphisms, C580**Y**, Y493**H**, R539**T**, and M476**L,** in propeller region of* K13* gene are strongly associated with artemisinin resistance. Following this discovery several workers reported a number of SNPs in this gene particularly at propeller region from different parts of the world [9, 19–26].

In the present study, we found that parasite was cleared in 53 cases on day 1 and in the remaining 6 cases on day 2. We did not find any mutation in the propeller part of the gene and reports of such mutation from this country are also rare [27]. However mutation at A578**S** which is adjacent to the C580**Y**, the major mutation causing delayed parasite clearance, was reported from neighboring country Bangladesh [22] and mutation at F446**I** from Myanmar [26].

## 5. Conclusion

ACT has been introduced in Kolkata just five years back. As we have not taken into account the parasite clearance half-life or ring stage survival assay in the study and the sample size is also small so it is difficult to comment on emergence of resistance against it. If the association of identified SNPs with artemisinin resistance is true, then this base-line information indicates that in urban Kolkata the prevailing* falciparum* population is still sensitive to artemisinin. Similar studies from different parts of the country and their association with delayed parasite clearance and RSA will be helpful to ascertain the emergence of artemisinin resistance, if any. Both quadruple and quintuple mutations in* pfdhfr *and* pfdhps *genes have been reported from different parts of the country [29]. As SP is used as partner drug of ACT, the in vivo efficacy study of this combination should be monitored at a regular interval of two years.

## Figures and Tables

**Table 1:** Primers and PCR conditions of primary and nested PCR of *K13* gene.

Primer name	Primer sequence (5′-3′)	Mg^2+^	PCR programme							
		Conc.	Denaturation		Annealing		Elongation		Number of cycles	
		(mM)	Temp.	Time	Temp.	Time	Temp.	Time	1st	2nd
			(°C)	(min)	(°C)	(min)	(°C)	(min)	PCR	PCR
K13_PCR_F K13_PCR_R	CGGAGTGACCAAATCTGGGA GGGAATCTGGTGGTAACAGC	3	94	0:30	60	1:30	72	1:30	40	—

K13_N1_F K13_N1_R	GCCAAGCTGCCATTCATTTG GCCTTGTTGAAAGAAGCAGA	2.5	94	0:30	60	1:30	72	1:30	—	40
